# Connecting to nature through community engaged scholarship: Community gardens as sites for collaborative relationships, psychological, and physiological wellbeing

**DOI:** 10.3389/fpsyt.2022.883817

**Published:** 2022-07-28

**Authors:** Kumara San Ward, Son Truong, Tonia Gray

**Affiliations:** ^1^Education and Society, School of Humanities, Social Sciences and Law, University of Dundee, Dundee, United Kingdom; ^2^School of Education, Western Sydney University, Penrith, NSW, Australia; ^3^School of Health and Human Performance, Dalhousie University, Halifax, NS, Canada

**Keywords:** community gardens, nature connectedness, physical and psychological wellbeing, community engaged scholarship, community partnerships

## Abstract

Community gardens are recognized as being associated with a range of benefits for participants that include enhanced outcomes in physical and affective domains and community building. The purpose of this study was to research the impact of the New South Wales Royal Botanic Gardens (RBG) Community Greening (CG) program and to inform the ongoing development of this community outreach program. The organic community partnerships inherent in the design and the relationships between the Community Greening program participants and researchers is examined through the lens of Community Engaged Scholarship (CES). Over a seven-month period, the CG team implemented a community garden development program in six sites. Mixed-method research on the impact of the program found that the community gardening participants experienced positive changes in physical activity, psychological wellbeing and motivation for social engagement, and these outcomes were facilitated as a result of their relationships with members of the CG team. This paper examines how such programs, when explicitly framed as CES, could assist in consolidating nature-based community health and wellbeing programs and further legitimize community partnerships in development of community garden and green spaces as academically sound investigation and socio-economically justified activity. Expansion of this nature-based collaboration model may also enhance community engagement in green exercise, psychological wellbeing and community cohesion, and in turn support advocacy for greener environments locally, regionally and nationally.

## Introduction

Community gardening is recognized for a range of benefits for participants which include supporting physiological, psychological and social wellbeing (1–4). In social housing contexts, community gardens can become sites for enhanced social connection, learning new skills, and for generating a sense of community pride (5) Additionally, they play a crucial role in place-making processes and generate the development of a sense of place while contributing to urban greening (6). This paper explores an example of community engaged scholarship through a study conducted in six new community gardens developed in social housing communities through the Sydney-based Royal Botanic Gardens and Domain Trust (RBG&DT), Community Greening (CG) program. Using a mixed method design, Western Sydney University academics engaged with the GC program coordinators to research the impact of these new community gardens sites in Western Sydney.

The CG program and associated research were developed with a focus on community-based partnerships and strengthening local participation. The structure and key intentions of the engagement between the GC program and Western Sydney academics reflect the concepts of Community Engaged Scholarship (CES) where community and academic interaction are nurtured, a culture of engaged citizenship with communities is a focus for academic institutions, and wherein pathways to career and/or academic success are enhanced (7). Therefore, the intention in this paper was to analyze the findings through a CES lens to highlight the role that the interactive community/agency and academic relationships played in the implementation and the outcomes of the program. The findings highlight participants' enhanced intra and interpersonal relationships and their role in fostering physiological and psychological wellbeing and community building. They include the perspectives of the management staff from the sites in which the gardens were situated, which correlate with those of the community garden participants.

These outcomes complement the findings from the original report (8) and two further papers where the focus is on community gardens as learning environments (5) and the benefits of urban greening in social housing (6). Finally, this paper recommends further research partnerships with communities between the academy, community organizations and community groups to facilitate genuine collaborations and to engage with knowledge and expertise held by community and community agencies.

The research questions underpinning this study were:

- What is the impact of participation in the Community Greening program on intrapersonal outcomes?
- What is the impact of participation in the Community Greening program on interpersonal outcomes?
- What are the participants perspectives of participation in the Community Greening program?

### Background to the community greening program

The Community Greening (CG) program was initiated in 1999 by the Royal Botanic Gardens and Domain Trust (RBG&DT) in Sydney, in collaboration with Housing New South Wales (NSW) (9). The program aimed to start outreach gardening programs in social housing developments serving low income and unemployed groups to support capacity-building, wellbeing and sustainable practices. Participants were mentored in building gardens, community management of gardens, learning horticulture, and in some settings, about identifying bush tucker. For over 20 years, the CG program has engaged with more than 100,000 participants, built 627 community gardens and aspires to increase this number to 150,000 participants and 850 community gardens in social housing areas within NSW by 2023. As such, there impact on wellbeing in social housing communities in the Greater Sydney area is significant.

### Wellbeing

Historically, one of the fundamental challenges of measuring wellbeing is the magnitude of disagreement over its definition and theoretical basis. However, there is universal consensus that wellbeing is multidimensional and clustered around the thematic domains of mental wellbeing, social wellbeing, physical/biological wellbeing, and spiritual wellbeing (10, 11). The perspective on wellbeing applied here is understood as dynamic and consisting of a range of domains, including individual, family, community, and societal (12, 13) and broadly reflects CG's approach to enhancing community wellbeing through gardening. The findings in this paper, encompass the above descriptions of wellbeing by reporting the outcomes through the impact of the intrapersonal, interpersonal and community relationships. This framework enables an in-depth understanding of how community members view and experienced community gardening, and its broader impact on health, wellbeing, and sense of community. It also provides a context to examine the nature of the engagement between the researchers, the CG program and the community participants, and the impact of the program, through the lens of CES.

The following section discusses the outdoor or green environment context of the CG program, an underpinning feature of the program as highlighted in the research findings. With this in mind, it is relevant to discuss the human/nature relationship, the implications for lack of access to natural or green environments, the benefits of community gardens, how they can affect psychological and physiological behavior and wellbeing.

## Related literature

### The human/nature relationship

Growing acknowledgment about human-nature interaction and its resultant impact on health promoting behaviors has consistently shown that contact with nature is vitally important for wellbeing (1, 14–18). However, such acknowledgment is concurrent with increased occurrence of loss of green space in urban areas (12, 19–22) thereby reducing the chances of regular nature contact among urban dwellers. Research on human-nature disconnection has highlighted subsequent negative effects on human health such as sedentary behavior, poor nutrition and social isolation (23–26). Human-nature connection also has an effect on behavior brain activity and mood states (27–29). To this end (30), articulate changes in behavior in terms of attitudes, beliefs and practices related to valuing of community green space and gardening, educational achievements, individual and collective problem-solving abilities and community identities. In this context, engagement in the natural world, through community gardening, has relevance as community education beyond the immediate benefits outlined above.

The increase in urbanization combined with a decline in green space has generated concerns in the past decade focusing on how it is affecting human wellbeing (26, 31–33). Researchers have argued that the innate connection with nature (34) has been disrupted in the recent times by densification of human populations and their activities (23, 35). Diminished green space encourages sedentary lifestyle in urban geographical areas intensifying the negative impact on individual, family and community wellbeing (36). In this context, wellbeing is understood as a dynamic process of multiple domains, including social, psychological, mental, emotional, environmental and physical, interacting with each other (37). Irregular and suspended contact with the natural world places at risk the interactional links between the wellbeing domains affecting humans from a holistic perspective (38). However, this effect may vary for different population groups depending on their access to engagement with the natural world.

### Access to nature

Contact with, or disconnection from nature in urban settings may be different for different population groups which raises questions pertaining to health equality and social justice (39, 40). In particular, urban low income population groups may be living with poor services being subjected to both social and environmental injustice (41). Braubach et al. (42) noted that urban lower socioeconomic population groups have limited access to green spaces often living in poorly serviced and neglected geographical patches of urban areas. They argued for reducing health inequalities in urban populations by improving the availability of green spaces in under-served and disadvantaged communities.

Population groups having refugee status are also often identified as low-income groups. Harris et al. (43) studied the participation of African refugees in a community gardening program to examine how they connected with their new homeland in Australia. They highlighted the community focus on building community connections and finding new opportunities of social connectedness. Various researchers have reported similar findings for low-income groups, but in addition include improvements in health and wellbeing, engagement in learning and social cohesion [For example: (3, 4, 44)]. These research studies, conducted with a multi-disciplinary approach, have affirmed the positive role of nature in human life cutting across gender, race, socio-economic status and cultural differences.

The urban landscape of metropolitan cities in Australia is rapidly changing reflecting similar global phenomenon of loss of urban green space (19, 22). Nonetheless, small patches of green features have the potential to grow social connectedness, reciprocity, respect and acceptance among community members (45). Porter (46) studied the participant experiences at a community food gardening program in the USA that was supported by community-based organizations, who were leading food justice movement in the country. Porter (46) raised important questions around “why and how gardening produces the outcomes” of improvements in health, enabling healing, growing food, and sustaining cultural ecosystem services in the community, and “for and with whom” (p. 14). Understanding these questions would, as she concluded, help in deepening and broadening the outcomes and sustain community gardening efforts further.

### Benefits of community gardens

Research has highlighted the physical, psychological and social health and wellbeing benefits of participating in gardening activities (1–4). For the purpose of this study, a community garden is defined as “an organized, grassroots initiative whereby a section of land is used to produce food or flowers or both in an urban environment for the personal use or benefit of its members” [(47), p. 79]. Community gardening programs address the lack of access to nature in many urban communities and the limited opportunities for engaging with it. The programs not only offer the residents a dedicated place to grow fresh food but also nurture in them a sense of belonging (8, 48).

Community gardens become sites for education, engagement and personal transformation for a wide range of issues. They support the cultivation of new behaviors, and provide motivation for residents to engage in regular physical activity, eat healthily and have an ongoing active social life with others in the community (48). This paper explores how the process of engaging in the development of community gardens supports physical and psychological wellbeing through fostering intrapersonal, interpersonal and community relationships and effective partnerships between community and the academy. The contexts of these activities and the relationships associated with them, are specifically related to outdoor engagement with gardens and nature. Therefore, the ways in which community garden environments can impact human behavior and relationships bears some consideration, particularly in relation to their potential to provide pleasurable outdoor engagement, foster social interaction and community-building.

Improved access to fresh food, better nutrition and improved mental health are some of the numerous benefits that community gardens offer to participants (1, 2). Research shows community gardeners feel proud of what and how they grow and share their joy in communal cooking activities, and in sharing their produce with others (49). It is these joyful positive feelings, along with increased physical activity (8), that can become features of psychological and physiological wellbeing beyond the engagement in the garden.

Community gardening initiates and fosters community bonding, social cohesiveness, and encourages group participatory process (15, 17, 30, 48). The gardens become expressive spaces to overtly show qualities such as cooperation, mutuality, sharing and caring for the resources and other community members (50). (47) adds that low socioeconomic groups may adopt gardens as their shared space for exchanges of cultural practices and also for acknowledging social capital. Continued participation and engagement in gardening may have prolonged impact on participants' improved health, wellbeing and social relatedness. In turn, these improvements have the potential to be perceived as motivating factors for life-long behavior change and, as has been argued above, a key reason for this may be the positive impact of collective human engagement with the natural world (51, 52). In the context of this study, the relationships with self and others that are nurtured throughout the process of building and maintaining the gardens also warrant examination. As this community gardening program was conducted in partnership with the CG team and with input from the researchers, an expanded view of the CES framework is a useful lens through which to consider the outcomes.

### Community engaged scholarship

Community Engaged Scholarship (CES) is defined by the Connecticut Campus Compact (CTCC) Engaged Scholarship Advisory Committee (2012) in the following way:

*Community engaged scholarship can be found in teaching, research and/or service. It is academically relevant work that simultaneously addresses disciplinary concerns and fulfills campus and community objectives. It involves sharing authority with community partners in the development of goals and approaches, as well as the conduct of work and its dissemination. It should involve critical review by discipline-specific peers, community partners and the public (p. 9)*.

The CTCC framework, is firmly underpinned by Boyer's (53) notion of scholarship where discovery (research), teaching, integration and application are the key pillars. The CTCC include descriptions, evaluation criteria and examples for CES as Service, Teaching, Research, and Reflection. They make a strong argument for higher education institutions to integrate CES into faculties so that community organizations and sites become places of learning and knowledge exchange that reflect the democratic “bottom up” approach to community development (30).

The notion of community in this research is consistent with McVey et al. (48), who conducted research into participant experiences of community gardens in Scotland. They characterize community as geographical and social with an emphasis on the place and group. Their work, while not specifically identified as CES, fulfills many of the tenets of CES through the researcher engagement with the community gardeners, the applied approaches recommended in their conclusions and the emphasis on beginning all processes for designing and implementing research with understanding of community needs and rights and in collaboration with communities.

The description of criteria for CES as service include: collaborative identification of issues with community partners to address a particular social issue, the work being subject to critical review and community peers, the work (and attendant findings) is publicly accessible and appropriately disseminated, there is evidence of impact, the work reflects good working relationships with community partners, and the work contributes to institutional and community capacity for engagement [(7), p. 19]. While the research study reported upon here was developed in consultation with CG, who in turn collaborated with community members, the oversight of the building of the gardens and the research were guided by a steering group with representatives from all stakeholders.

The criteria for considering CES as teaching include recognizing and using “service learning as a pedagogical tool to enhance faculty's teaching effectiveness” and “sharing insights about community impact, student learning and/or the teaching process with peers and colleagues to improve pedagogy in a field through the production of publicly-accessible scholarship” [(54), p. 27]. The teaching element in this study was more of a reciprocal exchange of knowledge where all participants were learning about the other and their expertise with a view to improving outcomes for all stakeholders. The scope for further engaging with communities and with undergraduate students is also considerable and is an element of CES that the researchers hope to develop in future.

Research and Reflection, as per the CES framework [(7), p. 19] refers to “scholarly collaboration with community partners which enacts, deepens understanding of, or creates knowledge within academic disciplines at the same time that it addresses community concerns” (p. 33) and argues “for an alternative definition of scholarship that recognizes the intersections of research, teaching, and service in the work” (42). CES recognizes that the expert knowledge may be beyond the academy and that dissemination and reflection may take a variety of forms to engage with the communities in which it is situated. These criteria are reflected through the WSU researchers collaborating in the design of the research, in engaging directly with the community and the CG partners and the CG team (the experts) in the building and planting of the gardens, in the social events that were held on the site days (shared community meals) and developing plans for dissemination. The intention in this study is to more fully characterize the work as CES with a view to recognizing and capitalizing on the potential benefits of doing so for all stakeholders in future engagement.

### Methodology

A pragmatic approach with mixed-methods design (55) was used to gain an in-depth understanding of the participant experiences generated from community gardening, consistent with co-constructionist and community work aimed at agency and transformation (56). The steering committee designing the research included key stakeholders from the communities, Family and Community Services, New South Wales (FACS, NSW), the RBG&DT, and a researcher from Western Sydney University. The research was conducted over a 12-month period (including planning, consultation and post research activities) and the research team visited the community gardens *in situ* over a period of 6 months. Two established and validated questionnaires (see below) were used in addition to focus groups and a questionnaire for community garden site staff, developed by the researchers. This approach enabled data to be triangulated within participant groups and across participant groups (55, 57).

#### Site selection and participants

The six separate community garden sites were invited to participate in the GC program as part of their outreach community engagement, therefore a purposive approach (55) to sampling was taken. They were located in linguistically and culturally diverse communities and three of the six sites were identified with an Index of Relative Socio-Economic Disadvantage being in the lowest 20% across Australia (58). Participation in the new community gardens was open to all site residents. All residents were invited to participate in the study related to the CG program through information posters and sessions at each site. Some residents chose not to participate but their decision had no impact on their access to or engagement in the gardens. A total of 53 participants from the six sites engaged in the research and four staff members from four sites. The specific numbers of participants from whom the different data sets were collected is outlined below.

#### Data collection

The study implemented two pre- and post-study questionnaires. The two questionnaires included the *Sense of Community Index 2* (59) and the *Personal Wellbeing Index* (60). The *Sense of Community Index 2* is the most frequently used quantitative measure of sense of community and casts sense of community as comprising four elements: membership, influence, meeting needs, and a shared emotional connection. All responses are in the form of rating scales. The *Personal Wellbeing Index* is an empirically validated scale that measures satisfaction across seven broad domains respectively: (1) standard of living; (2) health; (3) achievements in life; (4) personal relationships; (5) feelings of safety; (6) feeling part of the community; and (7) future security. There are also options in this instrument for limited qualitative responses.

The face-to-face focus group interview questions were semi-structured with flexibility promoting open-ended active discussion (61). A total of 42 participants across six sites participated in focus group interviews. Of these, 30 also completed the pre and post questionnaire. The remaining 12 focus group participants became involved with the community garden after construction of the garden beds. Out of the total, there were 26 females and 16 males who participated in the focus groups. The interviews ranged from 34 to 70 min in time duration, the average length being 50 min. Where possible the researchers encouraged participants to extend on their responses. In some instances, help from language interpreters was sought to assist in mutual interactions and effective communication. All the interviews were audio-recorded for later analysis.

Six months into the study staff members at all six gardening sites were provided with questionnaires by email. These staff members had varying roles and job titles in their communities ranging from community liaison to community development officer. However, they all had ongoing relationships with all members of the social housing estates in which they were employed, with the CG Program team and the research team, positioning them as participants in a CES framework (30). Their roles were significant in facilitating access, coordinating meetings and social events (lunches during building or gardening days) and as such they were key players in the relationship building process. Among a total of 15 questions, five sought responses on background information and community motivation for the construction of gardens, whilst 10 questions were related to the staff members' observations and perceptions on how the gardens impacted the participant behavior.

#### Data analysis

The focus group interviews were audio recorded and transcribed into texts. Both interview texts and the staff responses were uploaded to NVivo software to facilitate working with the data. Themes were developed through thematic coding (57) and analysis was guided by the constant comparative method (62, 63). Accordingly, initial codes were based on similarities and differences and contextually significant information identified in multiple readings of the texts, related to participant experiences and staff responses. The qualitative data—focus groups and *qualitative answers* in the questionnaires for participants and staff—are the focus of reporting in this paper. The rationale for this is to highlight the inherently subjective experiences shared by the participants and staff, which is best reflected through an interpretivist paradigm (61, 64). This re-analysis of the codes and themes originally developed, but now through CES lens, identifies the importance of relationships in impacting intrapersonal and interpersonal outcomes and on the role of the relationships inherent in community garden for community building. It involved ordering the established codes according to the number of references to them and refining the themes through the service, research and reflective lenses of CES. The outcomes echo the social constructivist nature of relationships in the community and those that are required for establishing community partnerships in CES (30).

The quantitative *Personal Wellbeing Index* contained limited options for qualitative responses and, where they relate to the qualitative data, have been included to further highlight layers of personal experiences that the participants valued allowing for deeper investigation of personal perspectives. These perspectives included information about the value of the community, CG team and academic relationships and provide an additional lens through which the inherent CES structures can be identified.

### Findings

#### Codes

The codes identified in the focus group transcripts are displayed in Table 1 and were drawn from 38 pages of text across the focus groups for the six sites. Responses are arranged below by codes, the number of times (references) the identified code was mentioned. The number of different sources from whom these references came are also displayed (sources).

**Table 1:** Focus group codes, references and sources.

	**Code**	**References**	**Sources**	**Words coded**	**Paragraphs coded**
1	Benefits—social engagement	53	10	3,610	146
2	Community—sense of community and inclusiveness	46	10	4,279	98
3	Community engagement development/aspirations and change of perspective	45	10	3,125	103
4	Benefits—wellbeing	39	10	2,140	86
5	Learning about gardening	39	10	4,096	130
6	Benefits Health, safety and security	35	8	4,040	120
7	Recommendations for improvement	34	10	3,055	54
8	Benefits, produce and cost	22	8	991	36
9	Favorite aspect	18	10	725	18
10	Barriers	17	6	908	12

There was a total of 18 codes from which the themes were generated. Those that were the most prominent are included in Table 1. Other codes and number of mentions included: Development of life skills-−15; Nature connection or environmentalism-−11; Intergenerational engagement-−10; Expertise and support-−8; Motivation and independent gardening-−7; intercultural features-−4; Motivation to garden-−3 and Planting and growing-−1. These additional codes are included narratively as some of them appear in participant quotes below. Those not included in quotes below are included for the sake of completeness.

#### Themes

The themes developed from focus group data indicate that involvement in the community garden is instrumental in enhancing social connection, inclusiveness and sense of community. Benefiting health and wellbeing, building personal social capital and supporting feelings of safety and security are also key themes. Finally, sense of community, encouraging aspirational change and changing stereotypes of public housing residents are discussed as key elements of participant responses. The findings below also integrate the participant data from the qualitative elements of the *Personal Wellbeing Index* and the narrative responses to the staff questionnaire. Together they indicate communities seeking to improve outcomes for all participants, to include participant voices and to build community for the benefit of all.

### Social connection, inclusiveness, and sense of community

Focus group participants articulated their belief that the community garden was instrumental for engaging with people in their local communities and for their communities forming a cohesive social group. The sites became a meeting place where social relationships were forged and nurtured and where everyone was included. In one focus group, participants described gardening as sharing culture and where the common commitment supported the forming of relationships that involved helping each other. These findings are reflected through the qualities of CES: The service element of CES is apparent in the development of good working relationships and contributing to the community capacity for engagement; the reflection and research elements are also reflected in the development of knowledge about each other which in turn may support the awareness and addressing of community concerns. The following quotes from focus group participants reflect this theme:

*Well, I know with me, I feel like I'm part of the community now, and I didn't feel like I was a part of it until now. (Resident Site 1)*

*Because there's a lot of people who have social anxieties and by creating a space for them to come out and have a chat, that decreases their social anxiety. (Resident Site 2)*

*I think this garden is big and beautiful, because everyone just work together and we can continue to grow this garden. And with this we live here in harmony and we cooperate with each other. I think it's very good for our community. Very good. (Resident Site 5)*

While the findings above come from the focus groups, the participants' qualitative responses in the *Personal Wellbeing Index* Questionnaire relate to the interpersonal elements of social engagement and being part of a community and provide a potential rationale for participants to continue to engage in the garden. When asked if coming to the community garden changed how satisfied they were with their personal relationships, residents written responses indicated they were more patient and they saw their neighbors more often. One also said that there were more differences of opinion becoming evident, giving rise to the need for understanding other perspectives and for developing negotiation skills. This recognition of differences of opinion was evident at some point in all of the communities and highlighted ephemeral or sometimes longer term tensions among residents. While this analysis does not seek to represent the outcomes as perfect, it does recognize that working with people from diverse linguistic, cultural and social groups can be complex and requires time and frequent opportunities for reflection and engagement.

The housing complex staff responded to a question asking about what they perceived to be the reasons for residents coming to the gardens by saying that they were learning new skills, feeling less stressed and actively enjoying growing, picking and eating fresh produce. Being able to share food and supporting harmony and cohesion were also mentioned as building social consciousness and pride in the community. These outcomes reflect the development of developing new expertise across stakeholder groups and further capacity for community engagement as per the service category of CES.

### Health and wellbeing, social capital, security, and safety

A number of categories from Table 1 relate to the findings regarding intrapersonal outcomes. These include benefits to wellbeing, health, produce and costs, development of life skills, nature connection, motivation to garden (and more generally), intercultural and intergenerational outcomes. The focus groups reported that the gardens provided a place of calm and introspection; they were almost meditational in addition to being a stress-free environment. They also identified satisfaction in making a contribution to the community and building their confidence in being part of a purposeful group who were engaged in positive activities. This development of individual health and wellbeing reflects the service elements of CES which highlights learning and teaching processes and sharing insights about the community. The participants also report enhanced levels of self-reflection and satisfaction with changes to their mental and physical health and wellbeing. They included becoming calmer when in the garden and an absence of depression and anxiety when working in the garden. One participant emphasized a change in their stress levels and identified a substantial change in their responses to difficult situations. Another commented that they had no anxiety or depression when working in the garden. They also identified a sense of community pride in seeing the appreciation of community members passing by the garden and sense of pleasure at their positive comments.

While the mental health benefits, including reduction in anxiety and stress, are evident from the descriptions above, the physical health benefits were also discussed with a focus on increased opportunities for physical activity through gardening. One participant even elaborated on their personal experience of improving their physical mobility to a point where they no longer used their walking cane over the course of the project due to improved physical activity levels.

*He wants me to be back on my cane. I don't want to be on my cane. I want to be independent on myself, and going and walking from there down to here, I can do it. (Resident Site 2)*

In contrast, the scores (rated between 1 not at all satisfied and 10 completely satisfied) on *The Personal Wellbeing Index* (60) questionnaire indicated that participants reported being marginally less satisfied with their health at post-test compared to pre-test (8). Although this may be concerning, a closer analysis reveals the age of the participants may have an impact on the capacity of community gardening to shift satisfaction with health. Differing markedly with this outcome, the tone and frequency of the qualitative comments from the focus groups, and the qualitative comments in the *Personal Wellbeing Index* questionnaire and those from the staff, indicate there were substantial changes in health outcomes for participants. When asked in the *Personal Wellbeing Index* Questionnaire if the community garden changed how satisfied they were with their health, the residents said that the garden made them happy, they were out in the sun and exercising and that they loved being there. Community site staff were asked about health and wellbeing impacts of the garden for the residents. They reported that they had observed improvements in mental and physical health and in one case increased confidence and fewer instances of social isolation for a resident with mental illness.

The final codes in this analysis related to health and wellbeing to produce and costs where participants were enjoying fresh food without having to pay for it, developing life skills through social negotiation and engagement with support services and building social capital and confidence. They also include nature connection through spending time outdoors, noticing the green areas of their neighborhoods or a new appreciation for sunset. The motivation to garden is also a feature which may be attributable to a number of intra and interpersonal benefits but which also reflect service elements of CES through contributing to the community and engagement.

### Sense of community, aspirations for improvement, and combating stereotypes

The strongest elements of community building that came through in the focus groups were the pride in community as a result of the garden projects and the relationships that were fostered in the community between community members, between community members and the CG team and to a lesser extent between community members and the researchers. Residents expressed satisfaction in being effective in changing community perspectives about people in social housing development referring to the changing of stereotypes and engaging with the broader community. Community members also talked about enhancing and enlarging their community garden or other areas within their community complexes. For one community this extended to a refurbishment of the community hall and for another it became engagement with the local council to protect an area of bushland adjacent to their community complex in order to maintain habitat for bees. The aspirations the participants had for changes to their communities were evident in their responses as was the desire to change the perception of social housing community residents to counter the stereotypes often attributed to people in low socio-economic circumstances. There are clear links to the impact of this CES partnership in these outcomes particularly to the elements of research and reflection where community knowledge is deepened and applied for the common good and in service elements of CES through the evidence of impact and the way in which the work of the CG program with the communities reflects good working relationships. The quotes below indicate the improvements recognized by participants and express the social cohesion and motivation to belong to community.

*So people slowly, slowly will start to exhibit what is called official “social cohesion”. I know it's a buzz word, but it works. (Resident Site 6)*

*Without a garden, you know, it would be just taking out the bins and checking the letterbox. But this gives you a reason to get together and spend a little bit of quality time. (Resident Site 3)*

When residents were asked in the *Personal Wellbeing Index* questionnaire if the community garden changed how satisfied they were with feeling part of their community, many responded affirmatively saying they were much more involved and could be with other people and develop new relationships, make genuine contributions and feel good about their communities. The housing community staff also responded about the ways in which the community garden helped to build a stronger community. They reported increased enthusiasm, cooperation, and an increase in social consciousness. They also reported that engagement in the community gardens strengthened relationships and provided additional opportunity for them to participate in the garden projects with residents, increasing trust as a result. This perspective of community capacity building demonstrates developing relationships where individual and community members work together with community agencies to achieve collaboratively developed aspirations and is clearly reflective of service in the CES construct.

One of the codes mentioned with the highest frequency (see Table 1) relates to learning about gardening. Every one of the 39 references to this code includes mention of the importance of the CG team. Positive relationships with the CG team are peppered throughout the focus group comments and while not part of the initial report analysis (8), these additional mentions about the CG team, across all codes, occur a minimum of 31 times. This highlights the value the participants placed on the engagement with the CG team, the steering group, and the researchers who worked with them, in developing the gardens and in providing ongoing workshops on different elements of gardening (workshops conducted by CG team), which is clearly reflective of the tenets of CES in service, teaching, research and reflection. These sentiments also highlighted the pleasure taken in learning about gardening and gratitude toward members of the CG team and their willingness to engage, listen and consult. Comments focused on the genuine engagement with the community members, the shared expertise, and the broader community advocacy in which the CG team supported the community members.

*But another way to get that feedback would be if [Community development officer] could also advise us through maybe* ^*^Alex on what works and what doesn't so that we can learn from other community development projects as well. (Resident Site 6)

*What I've learnt with* ^*^Alex and the with the other ladies here has just been so amazing, and I'm revitalizing my own garden at home and getting to grow more things again so it will be ongoing, and I'll just share it with my little grandchildren. (Resident Site 1)

NB: ^*^ indicates names changed for anonymity.

The community building discussion would be incomplete without mention of the events where all stakeholders were present. There were several construction days across the six sites, data collection days (pre and post garden building) and gardening workshop days where representatives from all groups were in attendance and engaged in the focus activity. These events were complimented by lunch or morning/afternoon tea services hosted by the communities and funded by the community organizations that supported the community housing sites. During these occasions there was a genuine sense of networking that contributed to community building where community participants and other stakeholders were able to discuss their engagement with each other with a view to developing new opportunities for mutual benefit, a key tenet of community engaged scholarship. As researchers involved in this process, we believed there were considerable opportunities for developing relationships with all stakeholders that could lead to community engagement for transformative action in the form of community engaged scholarship. Indeed, the inability to go beyond the remit of the limited research project and engage further with the communities was a source of frustration but also motivation to investigate other ways in which the various stakeholders could partner for further gain. This impetus led to the develop of a subsequent engagement with RBG/FACS and local communities to develop the Master Gardener Volunteer Program over a three-year period.

## Discussion

The intention in this paper was to analyze the qualitative data in the study through the lens of CES to identify the ways in which the relationships between partners and their community building activities could be conceptualized as community/agency/academic partnerships. Re-analysis of the qualitative data highlight the role of such partnerships in supporting social connection, inclusiveness and a sense of community that benefit health, wellbeing and development of personal and collective social capital and feelings of safety. The findings in these areas have been related to CES elements of service and research. They are analyzed below with a focus on the role of inter and intra relationships in fostering wellbeing and community building. The findings related to sense of community, aspirational change and challenging stereotypes has been identified in the CES elements of service, research and reflection and are analyzed below through a focus on community building.

### The role of inter and intrapersonal relationships in fostering wellbeing and community belonging

The changes to health and wellbeing reported by residents are consistent with McVey et al.'s (48) findings related to the social and leisure activities that communities engaged in as a result of their participation in gardens. Similarly, this study shows community members' health and wellbeing improving through sharing cultural knowledge such as recipes, and traditional approaches for food and mealtimes. Resident reports of a feeling of belonging are also relevant and significant. This sense of belonging, according to Kou et al. (30) is supported where there is an exchange of cultural knowledge and a shared collective memory. There are numerous reports of spontaneous socializing triggered by attendance at the gardens and a sense of belonging. Social anxieties are ameliorated through the opportunities for “chance engagements” in the gardens where the sense of community purpose to nurture the gardens relieves participants of the need for additional reason to be in the gardens, other than to garden. These activities are indicative of developing social capital both individually and collectively. Watson-Thompson (65) reminds us that a lack of social capital is one of the constraints that impedes communities from addressing the health and development issues that they face. Moreover, Iwasaki et al. (66) highlight the importance of social capital in their research with displaced communities due to the Fukushima disaster. Social capital gained through proximity to neighbors and engagement in community activities and volunteer work, played an important role in ameliorating distress and anxiety. With the development of improved individual health and wellbeing and reduced social anxiety being linked to improved individual social capital, it can be argued that the individual development of social capital contributes to collective social capital. The development of social capital supported by CG program reflects the service elements of CES through the impacts reported above but also reflect CES as research and reflection through the interagency, community and scholarly collaboration.

The positive intrapersonal outcomes for participants ranged from finding the garden as a stress-free zone, a meditational place, and a place where anxieties and depression were ameliorated, in addition to the physical health benefits. The types of mental health effects, as a result of being in green spaces and reported here, are also reported in the literature extensively, beginning with Kaplan's (67) work on attention restoration theory and the reduction of stress, up to the exploration of the effect of spending time in green spaces on executive function by Schutte et al. (68). The staff survey responses corroborate the self-reports by participants, indicating that they observed a change in residents. These changes included emerging from social isolation, increases in confidence and positive mood changes. The physical changes included increased mobility, eating more healthily and feeling joy in being active. (48) highlights the benefits of community gardens for improving the physical health of residents, particularly in light of the western world's propensity toward obesity where communities become more sedentary and rely more on convenience foods. Staff also report residents are markedly happier and that there are improvements to their physical health. Such changes in mental and physical health appear to be correlated with more engagement with others (30), including the CG team and the researchers, and are another indicator of the intrapersonal wellbeing benefits translating into improved social and community relationships. It is in these relationships that there is scope for further CES partnerships.

### The role of CES in community building

The CG Program team have been instrumental in building an enduring reputation over many years of community engagement as indicated in the background section of this paper. The networking among social housing community groups and their work with the Family and Community Services in New South Wales provided opportunities for outreach into new groups. In a manner similar to Kou et al. (30) in their work with five community gardens in Shanghai, the approach of the CG Program team is to engage with communities, not to impose services on them. One notable feature of the sites was the residents' diversity in cultural, linguistic and socio-economic factors, a cohort for whom service provision, rather than collaborative engagement and decision making, were more familiar. As McVey et al. (48) indicate, such diversity can become a community strength through which social capital is developed through the member relationships with a potential for social action beyond the gardens. A similar perspective on the importance of relationships and the role of trust was highlighted in an interview with the CG Coordinator at a new CG site in 2021, who encapsulated the respect and willing engagement the GC team have with the communities in which they work:

*So, basically, relationships are everything. It's about trust. A lot of it's to do with communities that have been let down a lot and have distrust of educational programs. Their whole formative learnings they've disengaged from often because they're kinaesthetic learners or they've had traumatic backgrounds. So, there is a big distrust of education full stop. So, bringing people back into engage in education is a really—you've got to tread very lightly. We work with community partners who've already been working with the community, and we develop a relationship with them. The programs that work really well are where Community Greening have actually been in those communities for a long time and they've developed trust and have developed those relationships beforehand: (*CG Program Coordinator, recorded conversation 2021).

The relationship building with the CG Program team and the researchers was approached from the perspective of the communities accepting the offer to create a community garden and to have genuine agency in its design, building and maintenance. The engagement of researchers from WSU was also underpinned by relationships built during previous research and community building projects and the researchers participated in the building of the gardens and in some of the follow up gardening education sessions with the residents. The relationships galvanized between the CG Program, researchers from WSU and their respective communities enabled the analysis of this study through the CES lens. In a similar manner to the roles that emerged for Kou et al. (30), in this study, the CG program were the experts, the gardening participants became knowledgeable networkers and co-learners, and the WSU, RBG and community agencies assumed the roles of facilitators and disseminators.

The key elements of the results related to community building are evident in the residents' comments related to caring for each other, having pride in themselves and their communities and having aspirations for further improvements in their gardens and communities. This is consistent with the finding of (48) who highlight the multiple roles that community gardens can play:

*The literature on community gardens highlights the role they can play in healthcare, learning, community-building and environmental stewardship, but many fail to examine community gardens as places of action that carry meaning beyond growing and the cultivation of food. A community garden could be an expression of the community it is in, the community that develops within the garden and the coordinators of the gardens themselves* (p. 44).

This is further emphasized in the residents' comments about wanting to change the view that the broader community has about them as housing estate residents. Their sense of belonging to a community is evident when they talk with pride about people from the broader community coming to look at the gardens. This highlights the sense of accomplishment in what they have collectively achieved and the collective social capital they have built (65). The number of residents comments about the engagement with the CG team, what they had learned from them about gardening and appreciation of their commitment to the gardens also highlights the importance of developing projects that come from “community identified initiatives of community-validated problems of social significance” [(65), p. 12].

The notion of community validated problems is significant when one considers the effects of self-claimed gardens in urban China (69). Zhu et al.'s work highlights that where there is no agreement for use of green space for community gardens tensions can arise that create community division rather than cohesion. However, in this study, the participants collaborated in the design of the gardens and entered into a partnership that also recognized the role of the researchers in reporting on the outcomes of the gardens as a mechanism for securing further funding and legitimacy for the ongoing support of their gardens and other potential projects funded under FACS. This commitment to building trust and rapport, and supporting community-based or community-led approaches evolved directly into development of a new partnership with RGB/FACS and members of the communities in this study and previous CG programs, to develop a new capacity building partnership through the Master Gardener Volunteer Program over a period of 3 years.

The community building outcomes are unmistakeably evident through the comments of the staff who describe the difference in motivation both for residents and themselves in participating in community garden building and workshop days and the social events that are integrated into them. They highlight the trust, increased cooperation and enthusiasm as being significant contributors to social cohesion and community building. Again, this is consistent with the literature (25, 30, 48, 65). The stakeholders in this study were part of the relationship building, opening new avenues for community engaged service and scholarship consistent with the aims of community building in a CES framework. The value of the relationships, as expressed by all participants in the CG program, shed a new light on CES and describe another way of recontextualising interventions in the outdoors with physiological and psychological impact. The nexus between the human/nature connection, the community relationships and enhanced wellbeing outcomes provide a potent recipe for building individual and collective social capital and ongoing partnerships.

The limitations of this study included the relatively small samples who completed the quantitative data sets and the implementation of only one focus group for each of the six garden sites after a seven-month period. Additional insights could have been gained through a small number of explanatory individual or dyadic interviews. There is also an inherent messiness in working with a number of partners where differences of opinion can have an impact on the smooth working of small groups. However, the richness and overall consistency of the qualitative data with participants from six sites provides a degree of credibility and qualitative trustworthiness (70).

## Conclusion

This paper has described a program by the Community Greening team from RBG&DT with multiple community housing sites in Western Sydney (NSW) in the process of building and maintaining community gardens over a seven-month period. The project was collaborative and interwoven with a broad range of perspectives from stakeholders during its inception, implementation and management. The research conducted of the program has been cast as community engaged scholarship with an acknowledgment that this was not an intended framework at the time the program and the associated research was conducted.

The focus of the study was finally positioned around the interpersonal, intrapersonal and community building impacts reported by the community residents and the staff engaged with the community housing developments. The analysis focused on the qualitative data from residents, and staff culminating in themes related to social connection, sense of community and community building. The findings highlighted the extent to which the CG program supported improved physical and mental wellbeing of participants, consistent with other findings on the benefits of community gardening (25, 30, 48) and the tenets of CES (65). The residents' responses and the underpinning literature on the human/nature relationship, also valorize the context of the program (i.e., engagement in a green space in a community garden) highlighting the effect of being in the outdoors for enhanced physical and psychological wellbeing outcomes. The ongoing thread of intra and interpersonal relationships form a strong nexus with respect to community and the development of social capital which is an integral component of CES.

The examination of this study through a CES lens has enabled a more critical focus that will support building on these outcomes with future research of this kind. Although it is clear the service, teaching and research elements of CES were present, future programs could involve additional focus on the voices of community participants in the identification of need for community action, design of the program, implementation, study design, and dissemination of findings, a process supported by Watson-Thompson's (65) recommendations for community engagement. Initial framing, using academically validated CES structures, may attract increased funding and engage the academy further in working with the community to progress the agenda for human/nature engagement for individual, collective community wellbeing and planetary health.

## Data availability statement

The datasets presented in this article are not readily available as permission was not sought during the ethics process to allow for sharing of same. Without said permission we are unable to provide access to the data. Requests regarding the datasets should be directed to kward001@dundee.ac.uk.

## Ethics statement

This study involved human participants and was reviewed and approved by the Human Research Ethics Committee: Western Sydney University. The participants provided their written informed consent to participate in this study.

## Author contributions

All authors contributed to design of the research, data collection, analysis, and contributed to creating text for the article and approved the submitted version.

## Funding

This research was commissioned by the New South Wales Department of Family and Community Services and the Royal Botanic Gardens and Domain Trust.

## Conflict of interest

The authors declare that the research was conducted in the absence of any commercial or financial relationships that could be construed as a potential conflict of interest.

## Publisher's note

All claims expressed in this article are solely those of the authors and do not necessarily represent those of their affiliated organizations, or those of the publisher, the editors and the reviewers. Any product that may be evaluated in this article, or claim that may be made by its manufacturer, is not guaranteed or endorsed by the publisher.
